# ^1^H NMR Metabolic Profile Discrimination of Three Monovarietal Olive Oils from Cultivars of the “Dauno” PDO Including Peranzana Young and Secular Tree Comparisons

**DOI:** 10.3390/molecules31132248

**Published:** 2026-06-26

**Authors:** Federica Angilè, Miriana Carla Fazzi, Chiara Roberta Girelli, Danilo Migoni, Francesco Paolo Fanizzi

**Affiliations:** Department of Biological and Environmental Sciences and Technologies, University of Salento, Prov.le Lecce-Monteroni, 73100 Lecce, Italy; federica.angile@unisalento.it (F.A.); mirianacarla.fazzi@unisalento.it (M.C.F.); chiara.girelli@unisalento.it (C.R.G.); danilo.migoni@unisalento.it (D.M.)

**Keywords:** olive oil, NMR spectroscopy, chemometric approach, multivariate statistical analysis, fatty acid profile, polyphenols

## Abstract

Olive oil represents a precious resource for the agri-food sector, not just economically but also for its health properties due to the presence of bioactive compounds. The aim of this study is to evaluate the Nuclear Magnetic Resonance (^1^H NMR)-based metabolomics profiles of monovarietal olive oils from Coratina, Ogliarola garganica and Peranzana, which are typically included in the “Dauno” PDO. Analysis by ^1^H NMR metabolic profiling was carried out to develop a strategy for understanding the differences based on cultivar. NMR spectroscopy coupled with multivariate statistical analysis (MVA) revealed significant differences in fatty acid profiles and phenolic compounds according to cultivar. In particular, Coratina oils were higher in squalene, oleic acid and polyphenols than Ogliarola garganica and Peranzana. Conversely, the latter two showed higher contents of polyunsaturated fatty acids and lower levels of phenolic compounds. Furthermore, as the olive oils were influenced by several factors, in addition to cultivar, the effects of tree age on the chemical composition of Peranzana olive oils were investigated. MVA revealed different fatty acid and polyphenol contents between secular and young trees. These results may contribute to expanding the literature data on the ^1^H NMR-based chemometric approach as a powerful tool for quality and authenticity control of olive oil.

## 1. Introduction

Extra Virgin Olive Oil (EVOO) represents one of the most important products in the agri-food sector in Mediterranean countries, as it forms the basis of the Mediterranean diet and is characterized by bioactive compounds responsible for specific health properties [1,2]. Despite the recent decrease, particularly in 2024, observed in the major countries contributing to the Italian region (Apulia) [3], Italy remains among the largest producers of the Mediterranean basin, along with Spain and Greece [4]. Interestingly, the reported production loss is due not only to *Xylella fastidiosa* infection [5] but also to prolonged drought periods, a primary consequence of climate change in southern regions [3]. In order to overcame the problem due to water availability and, at the same time, to satisfy the increase in olive oil demand, new strategies have been used recently. In particular, regulated deficit irrigation (RDI) treatment is a technique that allows for the reduction of water use during the phenological phase, which maintains crop quality and fruit yield [6]. Furthermore, the super high density system (SHD) is a new agronomic model introduced that provides olive growing with good economic sustainability [7]. These new approaches are expected to guarantee a high environmental sustainability [7], although the water availability in some specific regions such as Apulia cannot be overlooked [8]. Moreover, it should be noted that not all cultivars are suitable for these new agronomic strategies. At the same time, olive oil quality, with well-established chemical and sensory characteristics of autochthonous and traditional cultivars, has to be preserved. Therefore, it becomes important to find the right balance between the possible introduction of new cultivars, suitable for the RDI and/or SHD regime, and the preservation of product authenticity [7,9].

Moreover, other crucial questions are the geographical origin and traceability of olive oil [7,10,11]. Many studies demonstrated that quality as well as the chemical composition of olive oil depend on the growth area, climate, soil composition and water, in addition to the cultivar, fruit ripeness and harvest period [7,10,12]. Therefore, the authenticity and traceability of olive oil, including a geographical origin assessment, are issues that need to be carefully monitored, since the consumption of adulterated EVOO may represent, aside from economic fraud, a potential risk for consumer health [7,10]. In this regard, the European Union (EU) launched several government legislations and regulations [11,13,14], according to origin designation [7,11]. To date, there are several analytical methods to evaluate the authenticity and quality of olive oil, in particular in order to assign the specific commercial class, such as EVOO; however, the geographical origin assessment still lacks of an official validated methodology [1,10]. In this respect, Nuclear Magnetic Resonance (NMR)-based chemometric approaches have been largely used and proposed for olive oil cultivar, authenticity and/or geographical origin characterization [1,7,15,16]. Furthermore, this approach makes it possible to define the olive oil metabolic profiles while also considering different parameters, such as growing areas, pedoclimatic conditions and agricultural practices. These latter parameters may affect the product characteristics along with the genetic features of the used cultivars.

As reported in the literature, NMR spectroscopy is a non-destructive technique used for olive oil analysis due to the ease of sample preparation and rapid data acquisition [14]. NMR can be used to investigate both the major and minor components of olive oil, enabling a complete view of the natural variability of the sample’s chemical profile [1]. In recent years, numerous studies have been performed on olive oils using NMR spectroscopy coupled with multivariate statistical analysis (MVA). In particular, it has been possible to discriminate olive oil samples according to cultivar or growing area using NMR data and a chemometric approach, mainly Orthogonal Partial Least Squares Discriminant Analysis (OPLS-DA) or Linear Discriminant Analysis (LDA) [7,11,14,16,17,18,19,20,21]. In this work, NMR-based metabolomics analysis coupled with MVA was performed to better characterize monovarietal olive oils produced in Foggia, a province in the Apulia region, Southern Italy. Relevant cultivars currently in production and included in the “Dauno” PDO, one of the main Apulian PDOs, were analyzed [22]; in detail, Coratina, Ogliarola garganica and Peranzana were investigated. In addition to the differences among the three cultivars, attention was focused on the Peranzana variety. Peranzana is one of the popular cultivars of Foggia province, and it is sometimes considered as a “sweetener” that is often used in Coratina-based blends; however, to date, this cultivar is poorly characterized. In order to better characterize the Peranzana cultivar, the differences in the olive oil chemical profiles due to the age of the trees were evaluated. In fact, in addition to cultivar, climate, ripening stage and geographical origin, tree age also influences the chemical composition of olive oil [23,24].

## 2. Results

### 2.1. Multivariate Statistical Analysis of the Major Components of Coratina, Peranzana and Ogliarola Garganica Olive Oils 

As described in detail in the Section 4, two bucket datasets were generated from the NMR spectra. The stacked plot of representative ^1^H ZG NMR and ^1^H NOESYGPPS NMR results of olive oil is reported in Figure S1. In particular, BUCKET-1 was generated within the range of 10.00–0.5 ppm, in order to investigate the major components of olive oil, and BUCKET-2 was obtained within the range of 10.00–5.5 ppm, for the purpose of studying the unsaponifiable fraction.

First, an untargeted approach was performed on the main components to explore the dataset. A PCA model was obtained considering five principal components, with more than 85% of the explained variance, and R^2^X = 0.893 and Q^2^ = 0.814. In the PCA t[1]/t[2] score plot (Figure S2a), a clear separation among the samples was observed, according to cultivar. The separation among the oil samples according to the considered cultivars could be further improved by performing a supervised analysis with the class information, which yielded a significant PLS-DA score plot with reasonably good model parameters (4 components, R^2^X = 0.847, R^2^Y = 0.713, Q^2^ = 0.702). A visual inspection of the PLS-DA (Figure S2b) confirmed the separation among samples observed in the PCA model. In detail, Peranzana olive oils were well differentiated from Coratina and Ogliarola garganica olive oils, along the t[1] component, while Coratina and Ogliarola garganica samples resulted in separation along t[2] component. It should be noted that three samples of Coratina oil were excluded from the PLS-DA, as they resulted in outliers.

**Figure 1:**
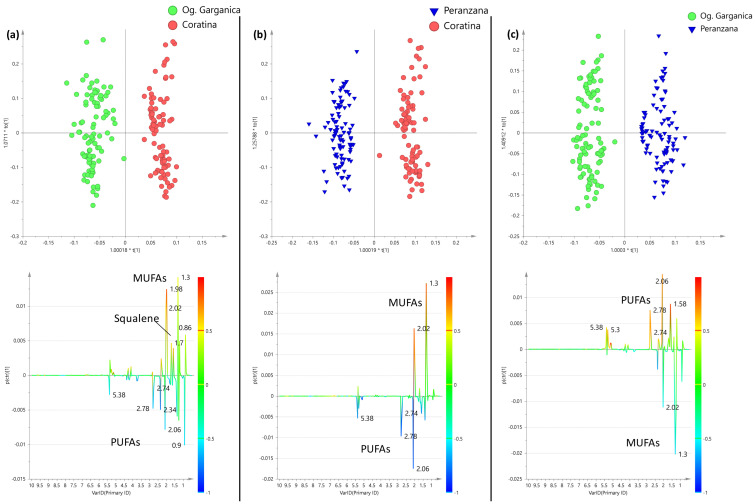
(**a**) OPLS-DA (1+3+0, R^2^X = 0.826, R^2^Y = 0.860, Q^2^ = 0.838) t[1]/to[1] score plot performed on major components (BUCKET-1) of olive oil samples obtained from Coratina and Ogliarola Garganica (**top**). S-line for the model (**bottom**). (**b**) OPLS-DA (1+3+0, R^2^X = 0.810, R^2^Y = 0.897, Q^2^ = 0.881) t[1]/to[1] score plot performed on major components (BUCKET-1) of olive oil samples obtained from Coratina and Peranzana (**top**). S-line for the OPLS-DA model (**bottom**). (**c**) OPLS-DA (1+3+0, R^2^X = 0.823, R^2^Y = 0.881, Q^2^ = 0.874) t[1]/to[1] score plot performed on major components (BUCKET-1) of olive oil samples obtained from Peranzana and Ogliarola Garganica (**top**). S-line for the OPLS-DA model (**bottom**). The variables indicate the chemical shift value (ppm) in the ^1^H NMR spectrum; Coratina (red circles), Ogliarola Garganica (green circles) and Peranzana (blue triangles).

In order to better and selectively evaluate the differences among the three studied cultivars, a supervised pairwise comparison was applied. As expected, the resulting OPLS-DA pairwise models (Figure 1) clearly account for the separation and comparison between each of the three possible couples of samples. By examining the loading S-line plot for each model, it is possible to identify the most influential variables (corresponding to the chemical shifts in the buckets that are representative of specific metabolites) responsible for sample discrimination in the pairwise comparisons. The loading S-line plot for the models reports the loading importance, colored according to the correlation-scaled vector (p(corr)). From the pairwise analyses, Coratina was characterized by a higher relative content of oleic acid (1.30 and 2.02 ppm) when compared separately to Peranzana and Ogliarola garganica. In addition, Coratina showed a higher level of squalene (1.7 ppm) in comparison with Ogliarola garganica, while Ogliarola garganica showed a higher relative content of oleic acid and linoleic acid (2.74 and 5.38 ppm) when compared with Peranzana and Coratina, respectively. Finally, Peranzana was characterized by a high PUFA (2.74, 2.78 and 5.38 ppm) content, in particular linoleic acid, with respect to both of the other cultivars.

The results observed in the pairwise comparisons were also confirmed by quantifying the variations in fatty acids among the three cultivars, which were calculated by the integration of unbiased signals and are reported in Table 1.

### 2.2. Multivariate Statistical Analysis of the Minor Components of Coratina, Peranzana and Ogliarola Garganica Olive Oils 

Despite fatty acids representing the major component in olive oils, the unsaponifiable fraction is very important when considering the health properties due mainly to the presence of phenolic compounds. For this reason, the minor components of the olive oils were also analyzed for the investigated cultivar samples using the same chemometric approach as for the major components. Therefore, multivariate statistical analysis was performed on the BUCKET-2 dataset, specifically related to the olive oil minor components.

A preliminary PCA was performed with the aim to observe the natural grouping of the samples. A good model was obtained with R^2^X = 0.776 and Q^2^ = 0.676, and five principal components described more than 75% of the total variance (t[1] and t[2] principal components explained 33% and 23%, respectively). The PCA model revealed a fair degree of separation based on the cultivars (Figure S3a). In order to refine the separation among the three olive cultivars, a supervised PLS-DA was performed including the class information (cultivar) of the samples. In the PLS-DA score plot (5 components, R^2^X = 0.743, R^2^Y = 0.756, Q^2^ = 0.719), the samples were again clearly separated (Figure S3b). In particular, the Coratina olive oil samples were clearly distinct with respect to the other two cultivars along the t[1] component, while Peranzana and Ogliarola garganica were separated along the t[2] component.

**Figure 2:**
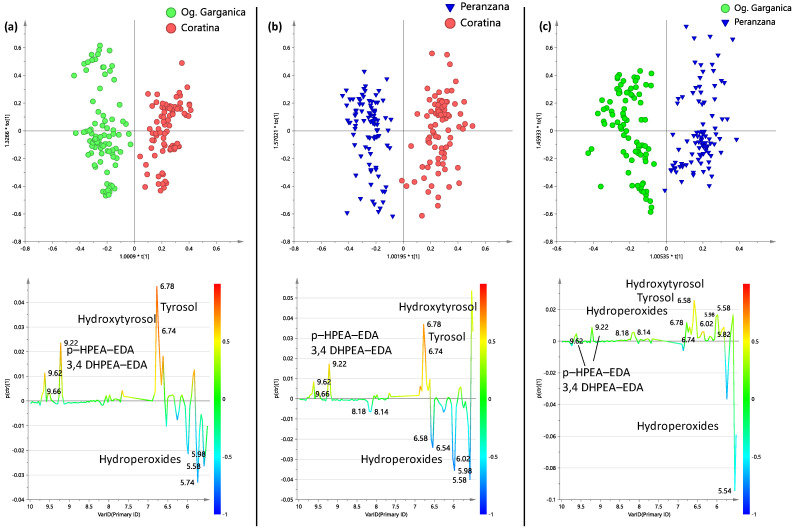
(**a**) OPLS-DA (1+3+0, R^2^X = 0.699, R^2^Y = 0.9 Q^2^ = 0.867) t[1]/to[1] score plot performed on minor components (BUCKET-2) of olive oil samples obtained from Coratina and Ogliarola Garganica (**top**). S-line plot for the model (**bottom**). (**b**) OPLS-DA (1+3+0, R^2^X = 0.702, R^2^Y = 0.899 Q^2^ = 0.862) t[1]/to[1] score plot performed on minor components (BUCKET-2) of olive oil samples obtained from Coratina and Peranzana (**top**). S-line for the OPLS-DA model (**bottom**). (**c**) OPLS-DA (1+3+0, R^2^X = 0.725, R^2^Y = 0.849 Q^2^ = 0.821) t[1]/to[1] score plot performed on minor components (BUCKET-2) of olive oil samples obtained from Peranzana and Ogliarola Garganica (**top**). S-line for the OPLS-DA model (**bottom**). The variables indicate the chemical shift value (ppm) in the ^1^H NMR spectrum; Coratina (red circles), Ogliarola Garganica (green circles) and Peranzana (blue triangles).

In order to better highlight the differences among the three studied cultivars, a series of OPLS-DA pairwise comparisons were also performed on the BUCKET-2 dataset. The results, presented in Figure 2, confirm that for all three possible comparisons, a good separation between the cultivars was achieved (Q^2^ ranging from 0.821 to 0.867). From the loading S-line plot analysis (Figure 2a,b), the Coratina cultivar was characterized by a higher relative content of polyphenols, such as tyrosol and hydroxytyrosol and their derivatives, in particular Oleacien (3,4-DHPEA-EDA) and Oleocanthal (p-HPEA-EDA), when compared with the Peranzana and Ogliarola garganica samples. The resonances at 6.74 ppm and 6.78 ppm were ascribed to protons in C-4 and C-7 of hydroxytyrosol and tyrosol and their derivatives, respectively. Furthermore, p-HPEA-EDA and 3,4-DHPEA-EDA exhibited NMR signals assigned at 9.22 ppm (aldehydic proton in C-1) and in the range of 9.66 ppm to 9.62 ppm (aldehydic proton in C-3). The oleocanthal (p-HPEA-EDA) signals were consistent with the literature data for the (3S,4E) diastereoisomer [25]. On the contrary, Peranzana and Ogliarola garganica olive oils showed a higher relative content of oxidation compounds, such as hydroperoxides, in contrast to Coratina olive oil.

In addition, the loading S-line plot analysis of the OPLS-DA model for the Peranzana and Ogliarola garganica comparison (Figure 2c) showed different relative contents of oxidation compounds between these two cultivars. In detail, Peranzana olive oil samples were characterized by a higher relative content of hydroperoxides associated with a (Z, E)-conjugated dienic system. The presence of this isomer was confirmed by the corresponding loadings (−CH=CH−CH=CH−) in the spectral range of 6.58–6.54 ppm; in contrast, olive oil obtained from Ogliarola garganica showed a higher relative content of hydroperoxides associated with a (E, E)-conjugated dienic system (loadings in the spectral range of 5.78–5.74 ppm, −CH=CH−CH=CH−). Moreover, Peranzana olive oil showed a higher level of phenolic compounds when compared to Ogliarola.

**Figure 3:**
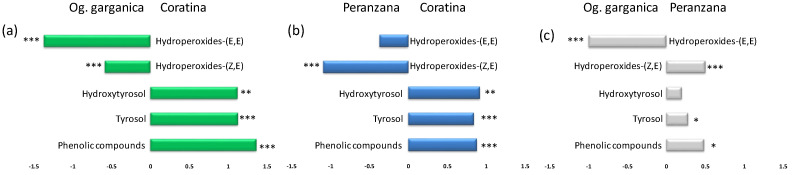
Variation in discriminating metabolites for Coratina, Ogliarola garganica and Peranzana cultivars. (**a**) Ogliarola garganica vs. Coratina; (**b**) Peranzana vs. Coratina; (**c**) Ogliarola Garganica vs. Peranzana. Signif. codes: 0 ‘***’ 0.001 ‘**’ 0.01 ‘*’ 0.05.

Furthermore, in addition to the results of the pairwise OPLS-DA comparison, the discriminating molecular components were also calculated from the mean values of the integrals of selected NMR resonances in the ^1^H NOESYGPPS spectra and are reported as −Log_2_FC (Figure 3). As observed in Figure 3a, a statistically significantly higher level of phenolic compounds in the Coratina cultivar with respect to the Ogliarola garganica and Peranzana cultivars was found. Moreover, Coratina showed significantly lower levels of hydroperoxides than the other cultivars, while a statistically significant difference, in particular for hydroperoxide isomers, was observed in the Peranzana versus Ogliarola garganica comparison (Figure 3b).

### 2.3. Multivariate Statistical Analysis of Peranzana Samples: Effect of Olive Tree Age

**Figure 4:**
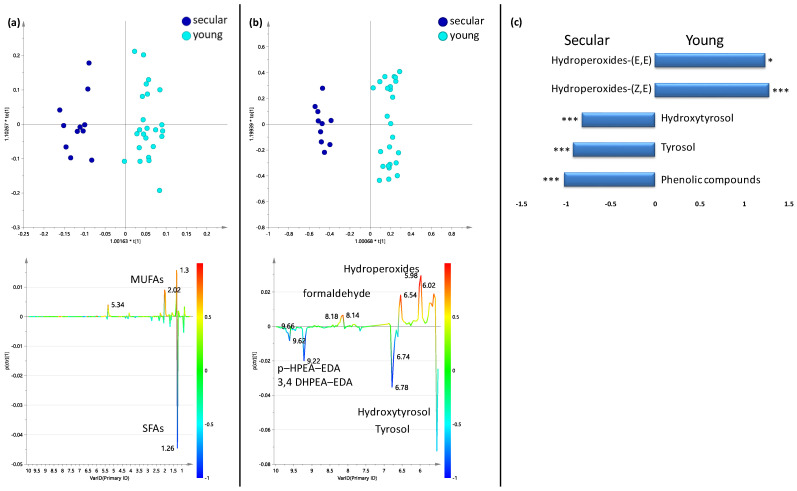
(**a**) OPLS-DA t[1]/to[1] score plot performed on major components (BUCKET-1) of olive oil samples obtained from secular and young trees of Peranzana cultivar (**top**). S-line for the model (**bottom**). (**b**) OPLS-DA t[1]/to[1] score plot performed on minor components (BUCKET-2) of olive oil samples obtained from secular and young trees of Peranzana cultivar (**top**). S-line line for the OPLS-DA model (**bottom**). (**c**) Variation in discriminating metabolites for Peranzana olive oil obtained from young and secular trees. Signif. codes: 0 ‘***’ 0.001 ‘*’ 0.05.

With the aim of obtaining more insights on the Peranzana olive oil, the olive tree age effects were also evaluated for this previously poorly investigated cultivar. In particular, Peranzana olive oil samples obtained in 2021 from secular (11 oil samples) and young trees (24 oil samples) were analyzed with multivariate statistical analysis. As observed from a visual inspection of the PCA and PLS-DA score plots (Figures S2 and S3), the olive oil samples obtained from secular (blue boxes) and young (blue stars) Peranzana trees were distributed according to the cultivar group, with no evidence of sampling sites. Therefore, by considering only Peranzana oil samples, an initial untargeted approach, followed by supervised analysis, was also achieved using the BUCKET-1 and BUCKET-2 datasets for the major and minor components. A PCA model was obtained, with 44% and 32% of the variance for the first, t[1], and second, t[2], components, respectively, and a predictive variation of Q^2^ = 0.711 (Figure S4). Observing the corresponding t[1]/t[2] PCA score plot, a nice separation of olive oil samples was revealed, mainly according to the age of the trees. The Peranzana olive oil sample separation was confirmed by the corresponding OPLS-DA (model obtained using one predictive and one orthogonal component 1+1, R^2^X = 0.757, R^2^Y = 0.916, Q^2^ = 0.867). As shown in the t[1]/to[1] score plot (Figure 4a), an unambiguous classification for the respective classes was found. The loading S-line plot for the model permitted the identification of metabolites responsible for the observed separation. In particular, the olive oil obtained from secular trees was characterized by a high relative content of saturated fatty acids, SFAs (1.26 ppm), while MUFAs (1.3 and 2.02 ppm), mainly oleic acid, were characteristic of the olive oil obtained from young trees. The percentages of the main fatty acids in olive oil obtained from young and secular trees were also directly calculated from the original NMR spectra and are reported in Table 2.

The same approach used for the major components was also performed on the unsaponifiable fraction, using the BUCKET-2 dataset. Similarly in this case, a preliminary PCA (Figure S5) showed a natural grouping of Peranzana oil samples based on the age of the trees. In detail, the PCA model was built considering three principal components, with more than 70% of the variance explained, R^2^X = 0.712 and Q^2^ = 0.572, with 47% and 17% variance for the t[1] and t[2] components, respectively. In order to confirm the separation between the oils obtained from secular and young plants, and to identify the discriminant metabolites, a supervised method was then applied (Figure 4b). In the OPLS-DA score plot of the model characterized by good parameters (1+2+0 components, R^2^X = 0.708, R^2^Y = 0.962, Q^2^ = 0.923), the olive oil samples obtained from secular trees were clearly differentiated from samples obtained from young plants along the predictive t[1] component. Examining the corresponding loading S-line plot for the model, it is evident that olive oil samples obtained from secular trees were characterized by high levels of polyphenol compounds, such as tyrosol, hydroxytyrosol, 3,4-DHPEA-EDA, and p-HPEA-EDA, with respect to oil samples obtained from young trees. These latter were characterized by higher relative contents of oxidation compounds and formaldehyde.

Furthermore, the variation in the discriminant metabolite content for Peranzana olive oils obtained from secular and young trees is reported as Log_2_(FC) (Figure 4c).

## 3. Discussion

In an effort to discriminate three monocultivar olive oils according to olive varieties, ^1^H NMR-based chemometric models were performed. Olive oils were obtained from popular cultivars in Foggia province (Apulia region, Italy); specifically, samples of Coratina, Ogliarola garganica and Peranzana were analyzed. Investigation by ^1^H NMR metabolic profiling and MVA was conducted to develop an approach for the accurate interpretation of differences based on cultivar. Furthermore, for Peranzana olive oils, unsupervised and supervised analysis also allowed the study of the discrimination based on olive tree age.

Interestingly, a good separation, according to cultivar, could be observed for the investigated samples even in the primary unsupervised PCA models using both the major and minor oil component-related, NMR-derived datasets (BUCKET-1 and BUCKET-2). A relatively high content of monounsaturated fatty acids, mainly oleic acid, and phenolic compounds, mainly represented, as already reported in the literature [26], by tyrosol, hydroxytirosol, p-HPEA-EDA (dialdehydic form of elenoic acid linked to tyrosol) and 3,4-DHPEA-EDA (dialdehydic form of elenoic acid linked to tyrosol), characterized Coratina compared to Ogliarola garganica and Peranzana olive oil samples. These results are in agreement with the literature data reporting higher oleic fatty acid and polyphenol contents for Coratina oils [1,26,27,28]. In fact, the Coratina variety is one of the most widespread and encouraged cultivars of Italy, in particular in the southern part of the country, with up to a 25% oil yield [29]. The high contents of oleic acid and phenols lend important beneficial health properties to Coratina olive oil. Specifically, oleic acid is very important for its crucial role in protection against several diseases, such as hepatic dysfunction and gut inflammation [1,10]. Furthermore, unsaponifiable components, such as tyrosol, hydroxytirosol, p-HPEA-EDA and 3,4-DHPEA-EDA, contribute to the sensory notes, the preservation of shelf-life and the olive oil’s nutritional/health characteristics [29]. Moreover, phenol compounds are responsible for the typical bitterness/pungent flavor of Coratina olive oil [1]. Due to the important bitter and pungent notes, Coratina olive oil is usually smoothed with other “sweetener” cultivar EVOOs, in order to obtain commercial blends [1]. As expected, because of their high total phenol content, Coratina oils also exhibit the highest resistance to oxidation [26,30]. In particular, because they are mainly composed of unsaturated fatty acid derivatives, such as oleic (18:1) and linoleic (18:2), EVOOs can be oxidized during storage and/or daily use [31]. Hydroperoxides represent the primary oxidation compounds in olive oil [6,32,33,34,35]. Due to the absence of a bis-allylic hydrogen, oleic acid is more resistant to radical oxidation than linoleic acid is. Therefore, the olive oil stability is strictly related to the oleic/linoleic ratio (ω-9/ω-6) [36]. This latter resulted in a value of 12.22 ± 2.56 for Coratina samples, a considerably higher value with respect to the 9.32 ± 0.93 found for Ogliarola garganica and 7.06 ± 1.30 for Peranzana. These data are consistent with the low content of hydroperoxides observed for Coratina oils, indicating a higher stability for this product with respect to the other oils investigated here. Furthermore, these results are in agreement with the literature data, in which Coratina olive oil showed a high antioxidant activity linked to the total polyphenol content (p-HPEA-EDA, 3,4-DHPEA-EDA), followed by Peranzana and Ogliarola olive oils [26]. Finally, Coratina olive oil also showed higher squalene levels in comparison with Ogliarola garganica. Squalene is a triterpene that is able to further improve the olive oil’s stability and therefore its shelf-life, and it is also known for its important health properties [1,2,37].

Concerning the Ogliarola garganica olive oil, despite it being a very common local cultivar, there are few studies that characterize this variety. Our results for these cultivars are in accordance with the literature data available for Ogliarola garganica. In particular, the obtained ω-9/ω-6 ratio of 9.32 ± 0.93 is similar to the value found by Del Coco, 9.43 ± 5.71 [36], suggesting a relatively good degree of stability for this cultivar in addition to the optimal rate found for Coratina olive oil. Furthermore, Del Coco reported that Ogliarola garganica olive oil samples were characterized by higher amounts of unsaturated fatty acids when compared with other cultivars that are widespread in Apulia, such as Ogliarola salentina and Cima di Mola [36]. Interestingly, this trend was also confirmed for the comparison of Ogliarola garganica with Coratina, with percentages of linoleic acid of 8.19 ± 0.65 and 6.77 ± 1.34, respectively. As expected, among the three studied cultivars, Ogliarola garganica exhibited minor contents of polyphenols, in particular, tyrosol, hydroxytyrosol, p-HPEA-EDA and 3,4-DHPEA-EDA identified by corresponded loadings of 6.78, 6.74, 9.22, 9.62 and 9.66 ppm, respectively. In fact, Ogliarola is generally considered among the “sweetener” cultivars due to the minor polyphenol amounts and the high content of saturated fatty acids [1,18]. Moreover, the analysis of the minor components of Ogliarola garganica oil showed a higher content of the isomer hydroperoxy-(E,E)-conjugated dienic system when compared to Peranzana samples. The hydroperoxy groups associated with the (E,E)-conjugated dienic system are derived from the oxidation of oleic acid [32,33,35]. This result is consistent with the oleic acid quantification, which was 76.06 ± 1.60 for Ogliarola garganica with respect to 74.04 ± 2.84 for Peranzana.

Among the cultivars investigated here, Peranzana olive oils showed the highest contents of PUFAs (mainly linoleic acid) and lower contents of MUFAs (oleic acid). Although a low content of oleic acid was previously reported for Peranzana olive oil [38], in the present work, the percentage of this key component (74.04% ± 2.84) was well over the minimum threshold for an effective nutritional effect [38]. Furthermore, Peranzana olive oil exhibited lower contents of polyphenols, such as tyrosol, hydroxytyrosol, p-HPEA-EDA and 3,4-DHPEA-EDA, mainly when compared with Coratina, suggesting its possible use as a “sweetener” cultivar. On the other hand, the Peranzana samples showed higher phenol contents, in particular tyrosol and its derivatives, with respect to Ogliarola garganica. Lastly, Peranzana oils showed a higher content of the hydroperoxidy-(Z,E)-conjugated dienic system. This latter isomer, the hydroperoxidy-(Z,E)-conjugated dienic system, originates from the oxidative degradation of linoleic acid [6,32,33,34], which exhibited the highest levels in Perananza samples (10.84 ± 1.94) compared to Ogliarola (8.19 ± 0.65) and Coratina (6.77 ± 1.34).

NMR-based chemometric models were also used here in an attempt to discriminate and better characterize Peranzana olive oils according to the age of the trees. Indeed, in addition to cultivar, storage conditions, harvest time and extraction method, the age of the trees also influences the chemical composition of olive oil, affecting the fatty acid and polyphenol profiles and consequently oxidative stability [39,40]. For this reason, Peranzana oil samples of the same harvesting year, specifically obtained in 2021, from young (San Paolo Civitate, Puglia, Italy) and secular (Torremaggiore, Puglia, Italy) trees were also compared in this work. Nowadays, there are limited literature data investigating the olive oil chemical composition according to tree age. In most studies, the effect of tree age on olive oil was also evaluated considering the variation in the other parameters, such as different harvesting times, altitudes and several pedoclimatic conditions [24,28,41,42,43].

In this study, the supervised models, obtained from both the major and minor component ^1^H NMR data (BUCKET-1 and BUCKET-2), revealed a good separation between olive oil samples according to tree age. In particular, with respect to the younger trees, higher relative contents of SFAs and linoleic acid were found in olive oil obtained from secular trees. The major content being SFAs in secular or adult trees in comparison with young trees is also observed in other cultivars, in particular the Chemali [23] and Oueslati varieties [24]. Furthermore, as reported by Bedbabis for Chemali, the increase in linoleic acid can be derived from the transformation of oleic into linoleic acid by oleate desaturase [23]. When considering the olive oil’s minor components, Peranzana olive oil obtained from secular trees showed a higher polyphenol content, in particular tyrosol, hydroxytyrosol, p-HPEA-EDA and 3,4-DHPEA-EDA, with respect to oil samples from young trees, suggesting a major oxidative stability. Nevertheless, as already discussed for the major components, the polyphenol amount in olive oil is also influenced by several factors such as the cultivar harvest time, storage conditions and geographical origin [7]. Our results are in agreement with previous literature data reporting a higher polyphenol content for olive oils of secular or adult trees in Oueslati and Picholine marocaine cultivars with respect to young trees [43,44]. Nevertheless, in other studies investigating the effect of olive tree age on the unsaponifiable fraction of the oil, a higher polyphenol content was observed in the olive oils obtained from young trees of Cellina di Nardò and Ogliarola salentina and Chemali [23]. Although the statistical significance of the results is partially affected by the small sample size, based on the results of the present work for Peranzana oils and the previous literature data, the polyphenol content differences according to tree age appear to be cultivar dependent. Thus, the reported data indicate the need for further research, which could focus on different cultivars, pedoclimatic conditions and olive-growing countries, in order to provide more general conclusions; these are beyond the scope of the present work. On the other hand, previous work from this group clearly defined the importance of harvesting year and the very fine differentiation of pedoclimatic condition effects when dealing with this kind of study [18,45,46]. Therefore, since the characteristics of the olive oil are also related to the specific harvesting year’s climatic conditions, this study should be repeated in subsequent years to understand and confirm the age-related results. Therefore, although these results are still preliminary, and further investigations are needed, the present data seem to show that at least for the considered geographical area and with the limitations of the model used, Peranzana olive oils exhibit an increase in the nutraceutically relevant polyphenol content in secular trees, consistent with reports for other cultivars.

## 4. Materials and Methods

### 4.1. Olive Collection and Oil Extraction

The olives of the Coratina (91 samples), Ogliarola garganica (96 samples) and Peranzana (95 samples) cultivars were collected in the 2021 and 2022 harvest years at orchards located in the municipalities of Torremaggiore and San Paolo Civitate, province of Foggia, belonging to the same geographical indication “Alto Tavoliere”; the areas have similar orographic and pedoclimatic characteristics, as stated by the specific production regulations of the Dauno Protected Designation of Origin (PDO) (Apulia Region, Italy) and are reported in Table 3 [22]. The olive samples were stored at −20 °C until the oil extraction. The olive oil was extracted by using laboratory-scale milling methods, as already reported by Angilè [7]. Briefly, about 50 olives were processed by fast-freezing with N_2_ and ground with a stainless-steel blender to obtain a paste. Thereafter, water was added, and the olive paste was stored at 4 °C overnight. Finally, the samples were centrifuged to obtain about 2–4 mL of oil, which was collected and stored until NMR analysis [7].

### 4.2. ^1^H NMR Spectroscopy Analysis

For NMR analysis, the olive oil samples were prepared according to the method already reported by Angilè [7]. In detail, about 140 mg of olive oil was dissolved in deuterated chloroform, CDCl_3_, containing tetramethylsilane, TMS, as an internal standard, at a proportion of oil:CDCl_3_ of 13.5:86.5 *w*/*w*. Then, 600 μL of the obtained mixture was transferred into a 5 mm NMR tube. The ^1^H NMR spectra were recorded on a Bruker Avance III 400 spectrometer (Bruker Italia, Milano, Italy) operating at 400.13 MHz, 300.0 K and equipped with a BBI 5 mm inverse detection probe incorporating a z-axis gradient coil. NMR spectrum acquisition was conducted in full automation for the whole process after loading individual samples on a Bruker Automatic Sample Changer (BACS) interfaced with IconNMR software version 5 (Bruker Italia, Milano, Italy). For each sample, automated tuning and matching, locking and shimming, and calibration of the 90° hard pulse P(90) were carried out by using the standard Bruker ATMA, LOCK; TOPSHIM and PULSECAL routines to maximize the NMR conditions. In order to characterize the fatty acid signals and to enhance the resonances of the minor components (i.e., polyphenols) for each sample, both standard ^1^H ZG NMR and a multi-suppressed ^1^H NOESYGPPS NMR (with suppression of intense fatty acid signals) experiments were performed [47,48,49]. Spectra were obtained with the following parameters: for ^1^H ZG, zg Bruker pulse program, 64 k time domain (TD), spectral width (SW) of 20.5524 ppm, a receiver gain (RG) of 4 and 16 number scans (NSs); for 1D NOESYGPPS NMR, noesygppps1d.comp2 Bruker pulse program, 32K TD, SW 20.5524 ppm, RG 16 and NS 32. ^1^H NMR spectra were obtained by the Fourier Transformation (FT) of the Free Induction Decay (FID), applying an exponential multiplication with a line broadening factor of 0.3 Hz, which was automatically phased and baseline corrected. Chemical shifts are reported with respect to TMS signals set as 0.0 ppm to obtain peak alignment. The metabolites were assigned on the basis of the ^1^H ZG and 1D NOESYGPPS spectrum analyses and by comparison with published data [6,7,21,25,35,50,51,52,53,54,55,56,57,58].

Fatty acid percentages were determined according to the simple methodology for fatty acid composition analysis in edible oils by NMR spectroscopy reported by Barison [6,59]. The fatty acid determination in olive oil samples was obtained directly from ^1^H NMR spectra without the use of complicated mathematical formulas. This method is based on the fact that the fatty acids are esterified to glycerol to form triglycerides. Since the signal area in the ^1^H NMR spectrum is proportional to the number of hydrogen atoms of each chemical group present in the sample, the fatty acid composition is determined through the relation between the area of the characteristic signal of a specific fatty acid chain and those of the glycerol backbone in the ^1^H NMR spectrum. In particular, the fatty acid percentages were evaluated by analyzing selected distinctive unbiased NMR signals. In detail, the signal areas at 4.29 ppm for the α hydrogen of the glycerol backbone, 0.98 ppm for the methyl hydrogen of linolenic, 2.74 ppm for the olefin hydrogen of linoleic, 2.02 ppm for the α olefin hydrogens of oleic and 2.28 ppm for the α carbonyl hydrogens of all fatty acids esterified to the glycerol moiety were considered. In this way, the percentage of a specific fatty acid was determined by integrating the characteristic resonance associated with its hydrogens relative to the integral of the glycerol backbone signal (4.29 ppm). Moreover, the ratio between the integrated signal area for the α hydrogen of glycerol and the specific fatty acid protons was converted into a percentage, and the glycerol signal was calibrated according to the resulting value. For example, the linolenic acid percentage was determined by integrating the signal at 0.98 ppm and comparing it with the signal area at 4.29 of glycerol. Considering that one glycerol molecule can bind up to three linolenic acid molecules, the corrected ratio is two α hydrogens of glycerol to nine methyl hydrogens of linolenic and corresponds to 22.2 (glycerol) to 100 (linolenic acid). Based on this reasoning, the percentages of fatty acids were calculated. The significance levels of the fatty acid compositions, obtained by the cited methodology, were higher than 95% for unsaturated and 91% for saturated fatty acids [59]. Further details on the fatty acid percentage calculation are provided in the Supplementary Materials (Figure S6, Table S1).

For phenolic compounds and peroxides, the signals corresponding to oleochantal/oleacein (9.22 ppm), tyrosol and derivatives (6.78 ppm), hydroxytyrosol and derivatives (6.74 ppm), hydroperoxy-(Z,E)-conjugated dienic system (6.58 ppm) and hydroperoxy-(E, E)-conjugated dienic system (5.74 ppm) were manually selected and integrated using TMS as well as the glycerol signals (4.33–4.26) as an internal standard. The significant differences in the mean values for the cultivars were obtained by analysis of variance (one-way ANOVA), with Tukey’s honestly significant difference (HSD) post hoc test, while for olive oil obtained from young and secular trees, the significant differences were obtained by a simple *t*-test. These test were performed using the R software package, version 4.4.2, on a 64 bit Windows machine (R, Development Core Team, 2013, Vienna, Austria) [60]. The level of statistical significance was a *p*-value < 0.05 with a 95% confidence level.

### 4.3. Data Processing and Multivariate Statistical Analysis

The 1D NMR spectra were processed using Topspin 3.6.5 and Amix 3.3.14 (Bruker, Biopspin, Italy), checked by visual inspection and then subjected to the bucketing process for multivariate statistical analysis (MVA) [7]. A rectangular bucketing of 0.04 width was performed in order to obtain two bucket tables in the 10.00–0.05 ppm region for ^1^H ZG (BUCKET-1) and the 10.00–5.50 ppm region for 1D NOESYGPPS (BUCKET-2), excluding the residual chloroform signals (7.6–6.9 ppm).

For all bucket row reduced spectra, total sum normalization was applied to minimize the small variation in the sample concentration and/or experimental conditions among samples. Furthermore, each bucket in a bucket row reduced spectrum was labelled with the central chemical shift values for its specific 0.04 ppm width. In the chemometric analysis, the input data matrix is represented by the samples’ NMR descriptors: the bucket row reduced spectra. A description of MVA refers to Pareto-scaled data obtained by dividing the mean centered bucket values by the square root of the standard deviation [7,61,62]. Both bucket tables, namely, BUCKET-1 and BUCKET-2, were obtained by alignment (using TMS for calibration at δ 0.00 ppm) and were separately submitted to MVA. Simca-14 software (Sartorius Stedim Biothec, Umeå, Sweden) was used for multivariate statistical analysis. In detail, unsupervised Principal Component Analysis (PCA) and supervised Partial Least Squares Discriminant Analysis (PLS-DA) and Orthogonal Projection to Latent Structures-Discriminant Analysis (OPLS-DA) were performed.

A PCA (the basis of the multivariate analysis) was applied to extract and display the systematic variation in a data matrix X formed by rows (the considered observation, in this case, olive oil NMR spectra) and columns (the variables that describe the samples, in our case, the buckets from each NMR spectrum) of the buckets from each NMR spectrum. The PCA model allows a general overview of all observations in the data table without class assignment to be obtained. The correlation between the cluster distribution of the analyzed samples and the considered classes (such as different varieties) is assessed by supervised MVA. PLS-DA is the main supervised method used for the discrimination between sample classes with different characteristics [1]. Furthermore, the OPLS-DA, used for the supervised MVA of the data, also included class information. The OPLS-DA represents a modification of the PLS-DA method, which, in addition to separating the sample groups according to both the original variables and the class information, filters out variation that is not directly related to the focused differentiation and produces models of clear interpretation. This results in the organization of the key discriminant information on one component, the predictive component, as already applied in different studies of metabolomics [63,64]. Indeed, the OPLS-DA has the ability to discriminate the portion of the variance that is useful for predictive purposes from the non-predictive variance (which becomes orthogonal). Statistical models were validated using the internal cross-validation default method (seven-fold) and further evaluated with a permutation test (one hundred permutations), all available in the SIMCA-14 software. Moreover, the R^2^ and Q^2^ parameters and CV-ANOVA were used to describe the quality of the model. In detail, R^2^ is a cross-validation parameter defined as the portion of data variance explained from the model and represents the goodness of fit. In addition, R^2^X and R^2^Y indicate the variance fraction of the matrix, X and Y, respectively. The usefulness of the model in predictions for class assignments of unknown samples is represented by the Q^2^ parameter. Since Q^2^ ≤ 1 and negative Q^2^ values indicate nonsignificant discriminations, values higher than 0.5 generally correspond to a good OPLS-DA model. In the supervised analysis, CV-ANOVA (cross-validated variance analysis) provides a *p*-value that describes the significance level of the group separation. Furthermore, the CV-ANOVA and permutation test were used to validate the predictive capacity and statistical significance of the OPLS-DA pairwise models. Moreover, CV-ANOVA provides a *p*-value below 0.05, and all permuted R^2^ and Q^2^ values with the intercepts of the R^2^ and Q^2^ values on the Y-axis, being <0.5 and 0, respectively, indicate a good fit for the OPLS-DA models. In addition, the Hotelling’s T-squared test, which is a generalization of Student’s *t*-test and defines, in conjunction with a score plot, the confidence area at 95 or 99%, were performed to identify strong outliers. Finally, the model’s predictive ability was further confirmed by the Fisher’s exact test using a misclassification table. The validation tests are reported in the Supplementary Materials [64,65,66]. In this work, the results are shown by the optimal by-dimensional score plots with the correspond loading plots; these latter plots were analyzed to identify the metabolites responsible for the separation among cultivars.

## 5. Conclusions

In the present work, Coratina, Ogliarola garganica and Peranzana monovarietal olive oils were analyzed by ^1^H NMR spectroscopy combined with multivariate statistical analysis (MVA). The analysis revealed that Coratina oils showed a higher content of MUFAs (oleic acid) with respect to Ogliarola garganica and Peranzana oil samples, which were richer in PUFAs (linoleic and linolenic acids). In addition, MVA showed high levels of polyphenols, such as tyrosol and hydroxytyrosol and their derivatives, in particular, oleocanthal (*p*-HPEA-EDA) and oleacein (3,4-DHPEA-EDA), in Coratina olive oils, which are related to the organoleptic characteristics of the same cultivar. On the contrary, olive oil samples obtained from Ogliarola garganica and Peranzana showed a peculiar profile distinctive of “sweetener varieties”, due to the lower content of phenolic compounds. Furthermore, a different metabolic profile for monovarietal Peranzana olive oil was observed within the same cultivar according to the age of the trees. Higher polyphenol, SFA and linoleic acid contents were found in secular versus young olive tree production.

Although the statistical significance of the results could be partially affected by the limited sample size and other factors such as seasonal effects, and while further investigations are needed, the present data seem to indicate that, at least for the considered geographical area, Peranzana olive oils exhibit an increase of the nutraceutically relevant polyphenol content in secular trees. However, further investigation, including a larger number of samples, could confirm the present descriptive results, thus improving the statistical reliability and validity of our outcomes.

## Figures and Tables

**Table 1:** Percentage of fatty acids by ^1^H ZG NMR spectra.

	% Fatty Acids				
Cultivar	Linolenic Ac.	Linoleic Ac.	Oleic Ac.	SFA	ω-9/ω-6
Coratina	1.11 ± 0.05 ^a^	6.77 ± 1.34 ^a^	79.32 ± 1.07 ^a^	12.80 ± 0.77 ^a^	12.22 ± 2.56 ^a^
Ogliarola Garganica	1.10 ± 0.06 ^a^	8.19 ± 0.65 ^b^	76.06 ± 1.60 ^b^	14.65 ± 1.25 ^b^	9.32 ± 0.93 ^b^
Peranzana	1.10 ± 0.11 ^a^	10.84 ± 1.94 ^c^	74.04 ± 2.84 ^c^	14.02 ± 1.37 ^c^	7.06 ± 1.30 ^c^

Different letters within the same column indicate significant differences among treatments for the fatty acids according to ANOVA with Tukey’s honestly significant differences (HSD) post hoc test; *p* value < 0.05.

**Table 2:** Fatty acid percentages calculated by integration of unbiased signals in the ^1^H ZG NMR spectra. Signif. codes: 0 ‘***’ 0.001 ‘*’ 0.05.

	% Fatty Acids				
Peranzana Samples	Linolenic Ac.	Linoleic Ac.	Oleic Ac.	SFA	ω-9/ω-6
Secular	1.03 ± 0.07	10.93 ± 0.87 *	73.73 ± 1.19 ***	14.32 ± 0.51 ***	6.79 ± 0.63 ***
Young	1.04 ± 0.05	10.28 ± 0.37	76.75 ± 0.71	11.93 ± 0.58	7.48 ± 0.31

**Table 3:** Pedoclimatic conditions of the sampling areas.

	Torremaggiore	San Paolo Civitate
Coordinates	41.6917 N, 15.2972 E	41.7393 N, 15.2611 E
Altitude (m a.s.l)	169	187
Altimetric zone	plain	plain
Mean annual temperature (°C)	16.0	16.1

## Data Availability

The original contributions presented in this study are included in the article/Supplementary Material. Further inquiries can be directed to the corresponding author.

## Supplementary Materials

The following supporting information can be downloaded at: https://www.mdpi.com/article/10.3390/molecules31132248/s1, Figure S1. Stacked plot of a representative ^1^H ZG NMR (blue, bottom) and ^1^H NOESYGPPS NMR spectrum (black, upper) of olive oil in CDCl_3_ obtained at 400 MHz. The main resonances (NMR signals) responsible for the olive oil separation are marked in the spectrum, TAGs: triglycerides. Figure S2. (**a**) t[1]/t[2] PCA score plot, 5 PCs R^2^X = 0.893 and Q^2^ = 0.814, performed on major components (BUCKET-1) of olive oil samples obtained from Coratina, Ogliarola garganica, and Peranzana; (**b**) t[1]/t[2] PLS-DA score plot, 4 components, R^2^X = 0.847, R^2^Y = 0.713, Q^2^ = 0.702, performed on major components (BUCKET-1) of olive oil samples obtained from Coratina, Peranzana, and Ogliarola Garganica. Coratina: pink circles; Ogliarola garganica: green circles; Peranzana: blue triangles (Peranzana samples obtained from secular: blue box; Peranzana samples obtained from young: blue star); (**c**) Permutation test performed with 100 cycles of random permutation of Y variables on PLS-DA obtained for major components of olive oils (BUCKET-1). The horizontal axis shows the correlation between the original and permuted y. The vertical axis shows the values for R2 (green line) and Q2 (blue line). The intercept is a measure of the overfit. A steep slope indicates well fitted. Table CV-ANOVA obtained for PLS-DA model. Figure S3. (**a**) t[1]/t[2] PCA score plot, 5 PCs R^2^X = 0.794 and Q^2^ = 0.688, performed on minor components (BUCKET-2) of olive oil samples obtained from Coratina, Ogliarola garganica, and Peranzana; (**b**) t[1]/t[2] PLS-DA score plot, 5 components R^2^X = 0.743, R^2^Y = 0.756, Q^2^ = 0.719, performed on minor components (BUCKET-2) of olive oil samples obtained from Coratina, Peranzana, and Ogliarola Garganica. Coratina: pink circles; Ogliarola garganica: green circles; Peranzana: blue triangles (Peranzana samples obtained from secular: blue boxes; Peranzana samples obtained from young: blue stars); (**c**) Permutation test performed with 100 cycles of random permutation of Y variables on PLS-DA obtained for major components of olive oils (BUCKET-2). The horizontal axis shows the correlation between the original and permuted y. The vertical axis shows the values for R^2^ (green line) and Q^2^ (blue line). The intercept is a measure of the overfit. A steep slope indicates well fitted. Table CV-ANOVA obtained for PLS-DA model. Figure S4. t[1]/t[2] PCA score plot, 5 PCs R^2^X = 0.926 and Q^2^ = 0.711, performed on major components (BUCKET-1) of olive oil samples obtained from young and secular trees of Peranzana. Figure S5. t[1]/t[2] PCA score plot, 3 PCs R^2^X = 0.712 and Q^2^ = 0.572, performed on minor components (BUCKET-2) of olive oil samples obtained from young and secular trees of Peranzana. Figure S6. ^1^H ZG NMR spectrum of olive oil with respective assignment of the signals of the glycerol and the fatty acids. Table S1. Summary of the methodology developed to determine the fatty acid composition in edible oils by Barison et al. Figure S7. Permutation test performed with 100 cycles of random permutation of Y variables on OPLS-DA obtained for major components of olive oils (BUCKET-1). The horizontal axis shows the correlation between the original and permuted y. The vertical axis shows the values for R^2^ (green line) and Q^2^ (blue line). The intercept is a measure of the overfit. A steep slope indicates well fitted. Table CV-ANOVA obtained for OPLS-DA model: (**a**) Permutation test and CV-ANOVA for OPLS-DA model of olive oil samples obtained from Coratina and Ogliarola Garganica; (**b**) Permutation test and CV-ANOVA for OPLS-DA model of olive oil samples obtained from Coratina and Peranzana; (**c**) Permutation test and CV-ANOVA for OPLS-DA model of olive oil samples obtained from Ogliarola garganica and Peranzana; (**d**) Permutation test and CV-ANOVA for OPLS-DA model of olive oil samples obtained from young and secular trees of Peranzana. Figure S8. Permutation test performed with 100 cycles of random permutation of Y variables on OPLS-DA obtained for minor components of olive oils (BUCKET-2). The horizontal axis shows the correlation between the original and permuted y. The vertical axis shows the values for R^2^ (green line) and Q^2^ (blue line). The intercept is a measure of the overfit. A steep slope indicates well fitted. Table CV-ANOVA obtained for OPLS-DA model: (**a**) Permutation test and CV-ANOVA for OPLS-DA model of olive oil samples obtained from Coratina and Ogliarola Garganica; (**b**) Permutation test and CV-ANOVA for OPLS-DA model of olive oil samples obtained from Coratina and Peranzana; (**c**) Permutation test and CV-ANOVA for OPLS-DA model of olive oil samples obtained from Ogliarola garganica and Peranzana; (**d**) Permutation test and CV-ANOVA for OPLS-DA model of olive oil samples obtained from young and secular trees of Peranzana. Figure S9. (**a**) Hotelling’s T^2^ Range plot for PCA model performed on major components (BUCKET-1) of olive oil samples obtained from Coratina, Ogliarola garganica, and Peranzana; (**b**) Hotelling’s T^2^ Range plot for PCA model performed on major components (BUCKET-1) of olive oil samples obtained from young and secular trees of Peranzana; (**c**) Hotelling’s T^2^ Range plot for PCA model performed on minor components (BUCKET-2) of olive oil samples obtained from Coratina, Ogliarola garganica, and Peranzana (**b**) Hotelling’s T^2^ Range plot for PCA model performed on minor components (BUCKET-2) of olive oil samples obtained from young and secular trees of Peranzana. Figure S10. Hotelling’s T^2^ Range plot for the OPLS-DA obtained for major components of olive oils (BUCKET-1). (**a**) Coratina and Ogliarola garganica comparison; (**b**) Coratina and Peranzana comparison; (**c**) Peranzana and Ogliarola garganica comparison; (**d**) young and secular trees of Peranzana. Figure S11. Hotelling’s T^2^ Range plot for the OPLS-DA obtained for minor components of olive oils (BUCKET-2). (**a**) Coratina and Ogliarola garganica comparison; (**b**) Coratina and Peranzana comparison; (**c**) Peranzana and Ogliarola garganica comparison; (**d**) young and secular trees of Peranzana. Table S2. Misclassification table (SIMCA-P software Version 5) obtained for OPLS-DA model analysis performed for major components (BUCKET-1) and minor components (BUCKET-2) of olive oils obtained from young and secular trees.

## Abbreviations

The following abbreviations are used in this manuscript:

NMR	Nuclear Magnetic Resonance Spectroscopy
MVA	Multivariate Statistical Analysis
PCA	Principal Component Analysis
PLS-DA	Partial Least Squares Discriminant Analysis
OPLS-DA	Orthogonal Projection to Latent Structures-Discriminant Analysis
SFA	Saturated Fatty Acid
PUFA	Polyunsaturated Fatty Acid
MUFA	Monounsaturated Fatty Acid
PDO	Protected Designation of Origin
