# Supplementary material for: 1H NMR Metabolic Profile Discrimination of Three Monovarietal Olive Oils from Cultivars of the “Dauno” PDO Including Peranzana Young and Secular Tree Comparisons
Source: Molecules. 2026 Jun 26;31(13):2248. doi: 10.3390/molecules31132248 (PMC13362912; doi:10.3390/molecules31132248)
Supplement: Supplementary file 1 [file molecules-31-02248-s001.zip › molecules-4221276-supplementary.pdf]

^{1}H NMR Metabolic Profile Discrimination of Three Monovarietal Olive Oils from cultivars of the "Dauno" PDO including Peranzana young and secular tree comparison

Federica Angilè, Miriana Carla Fazzi, Chiara Roberta Girelli, Danilo Migoni, Francesco Paolo Fanizzi*

Department of Biological and Environmental Sciences and Technologies, University of Salento, Prov.le Lecce-Monteroni, 73100 Lecce, Italy

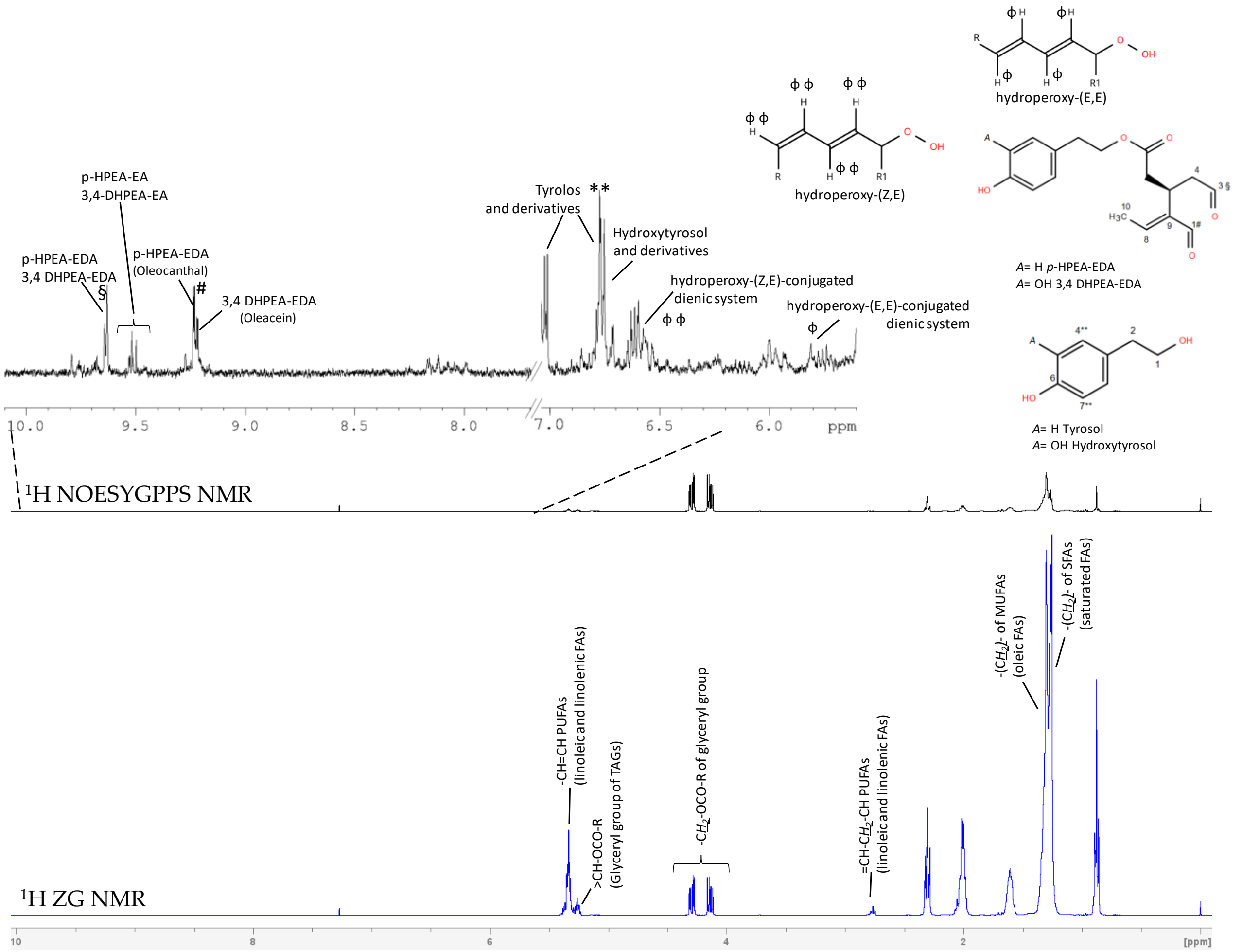

Figure S1. Stacked plot of a representative ^{1}H ZG NMR (blue, bottom) and ^{1}H NOESYGPPS NMR (black, up) spectra of olive oil in $CDCl_3$ obtained at 400MHz. The main resonances (NMR signals) responsible for the olive oil separation were marked in the spectrum. TAGs: triglycerides.

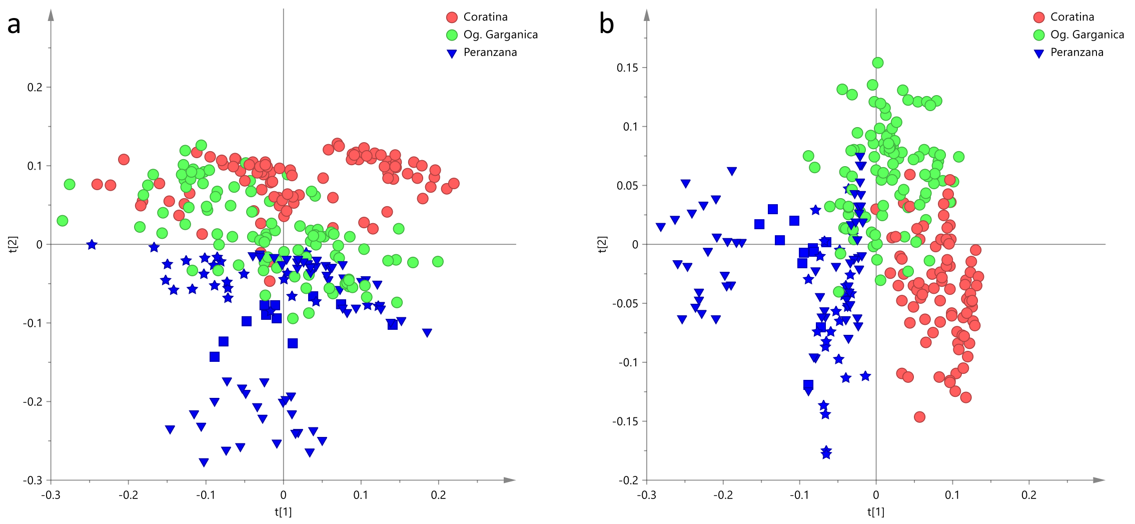

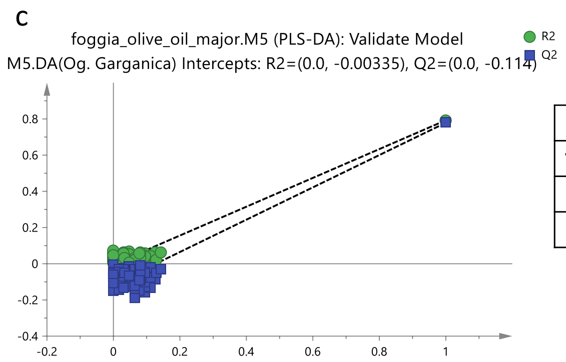

M5	SS	DF	MS	F	p	SD
**Total corr.**	556	556	1			1
**Regression**	420.309	16	26.2693	104.542	0	5.12536
**Residual**	135.691	540	0.251279			0.501277

Figure S2.a) t[1]/t[2] PCA score plot, 5 PCs $R^2X=0.893$ and $Q^2=0.814$, performed on major components (BUCKET-1) of olive oil samples obtained from Coratina, Ogliarola garganica, Peranzana; **b)** t[1]/t[2] PLS-DA score plot, 4 components, $R^2X=0.847$, $R^2Y=0.713$, $Q^2=0.702$, performed on major components (BUCKET-1) of olive oil samples obtained from Coratina, Peranzana, Ogliatola Garganica. Coratina: pink circle; Ogliarola garganica: green circle; Peranzana: blue triangle (Peranzana samples obtained from secular: blue box; Peranzana samples obtained from young: blue star); **c)** Permutation test performed with 100 cycles of random permutation of Y variables on PLS-DA analysis obtained for major component of olive oils (BUCKET-1). The horizontal axis shows the correlation between the original and permuted y. The vertical axis shows the values for R^2 (green line) and Q^2 (blue line). The intercept is a measure of the overfit. A steep slope indicates well fit. Table CV-ANOVA obtained for PLS-DA model.

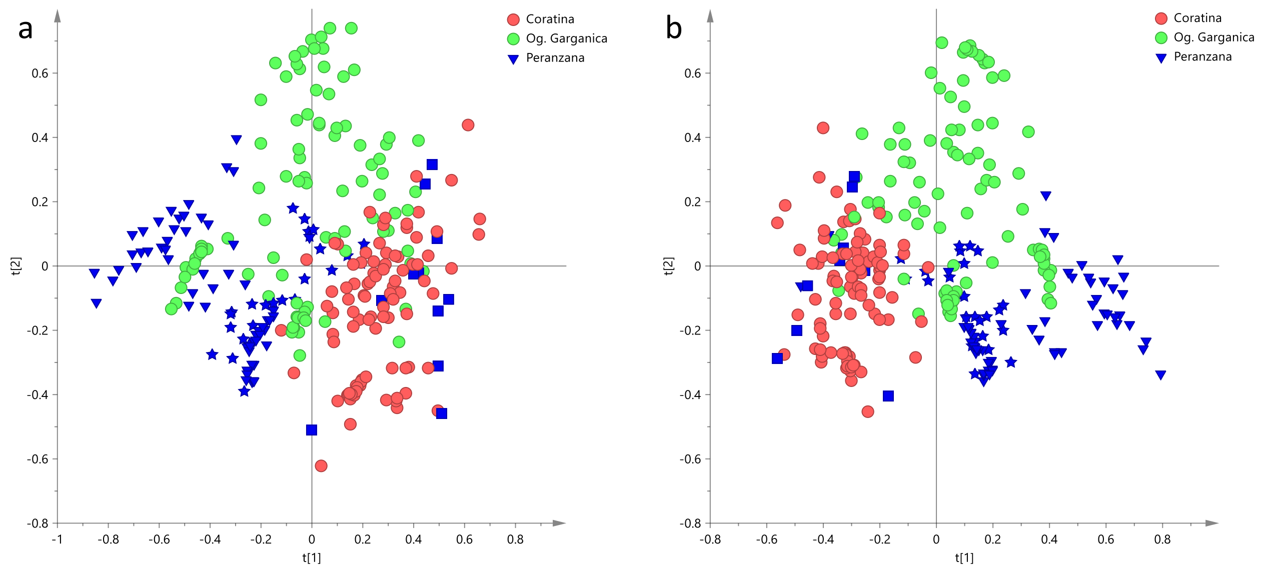

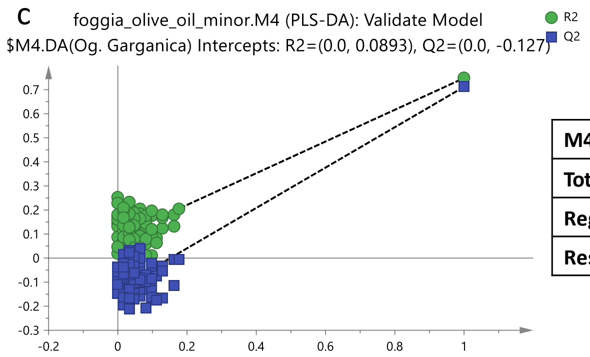

M4	SS	DF	MS	F	p	SD
**Total corr.**	556	556	1			1
**Regression**	371.136	20	18.5568	53.8043	0	4.30776
**Residual**	184.864	536	0.344895			0.587277

Figure S3. **a)** t[1]/t[2] PCA score plot, 5 PCs $R^2X=0.794$ and $Q^2=0.688$, performed on minor components (BUCKET-2) of olive oil samples obtained from Coratina, Ogliarola garganica, Peranzana; **b)** t[1]/t[2] PLS-DA score plot, 5 components $R^2X=0.743$, $R^2Y=0.756$, $Q^2=0.719$, performed on minor components (BUCKET-2) of olive oil samples obtained from Coratina, Peranzana, Ogliarola Garganica. Coratina: pink circle; Ogliarola garganica: green circle; Peranzana: blue triangle (Peranzana samples obtained from secular: blue box; Peranzana samples obtained from young: blue star); **c)** Permutation test performed with 100 cycles of random permutation of Y variables on PLS-DA analysis obtained for major component of olive oils (BUCKET-2). The horizontal axis shows the correlation between the original and permuted y. The vertical axis shows the values for R^2 (green line) and Q^2 (blue line). The intercept is a measure of the overfit. A steep slope indicates well fit. Table CV-ANOVA obtained for PLS-DA model.

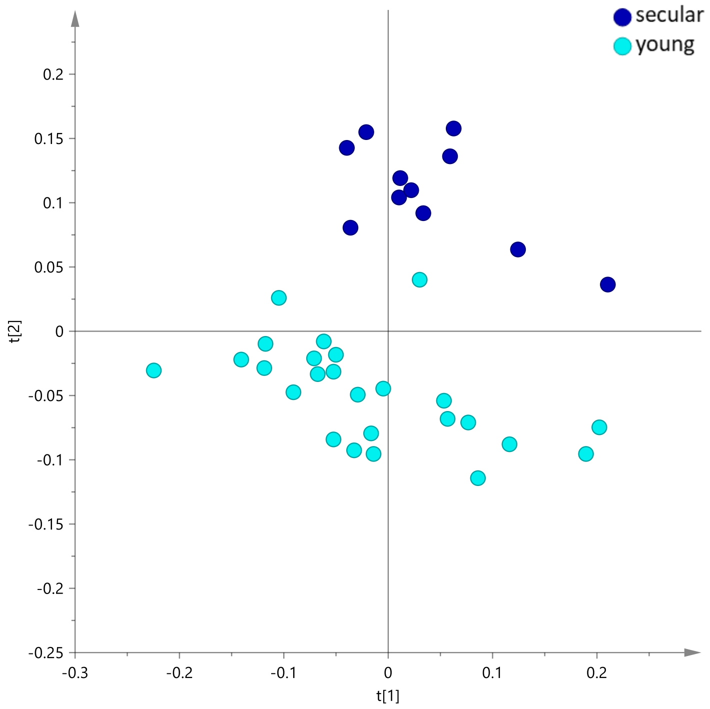

Figure S4. t[1]/t[2] PCA score plot, 5 PCs $R^2X=0.926$ and $Q^2=0.711$, performed on major components (BUCKET-1) of olive oil samples obtained from young or secular trees of Peranzana.

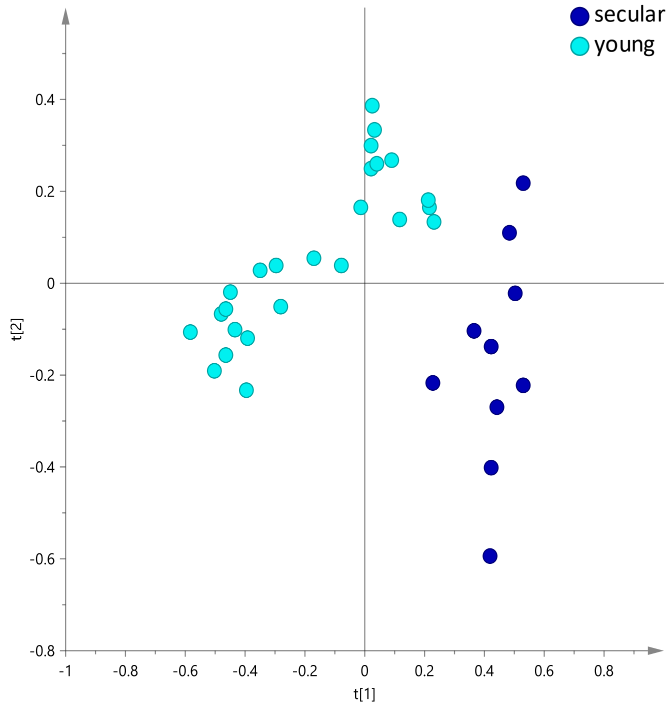

Figure S5. t[1]/t[2] PCA score plot, 3 PCs $R^2X=0.712$ and $Q^2=0.572$, performed on minor components (BUCKET-2) of olive oil samples obtained from young or secular trees of Peranzana.

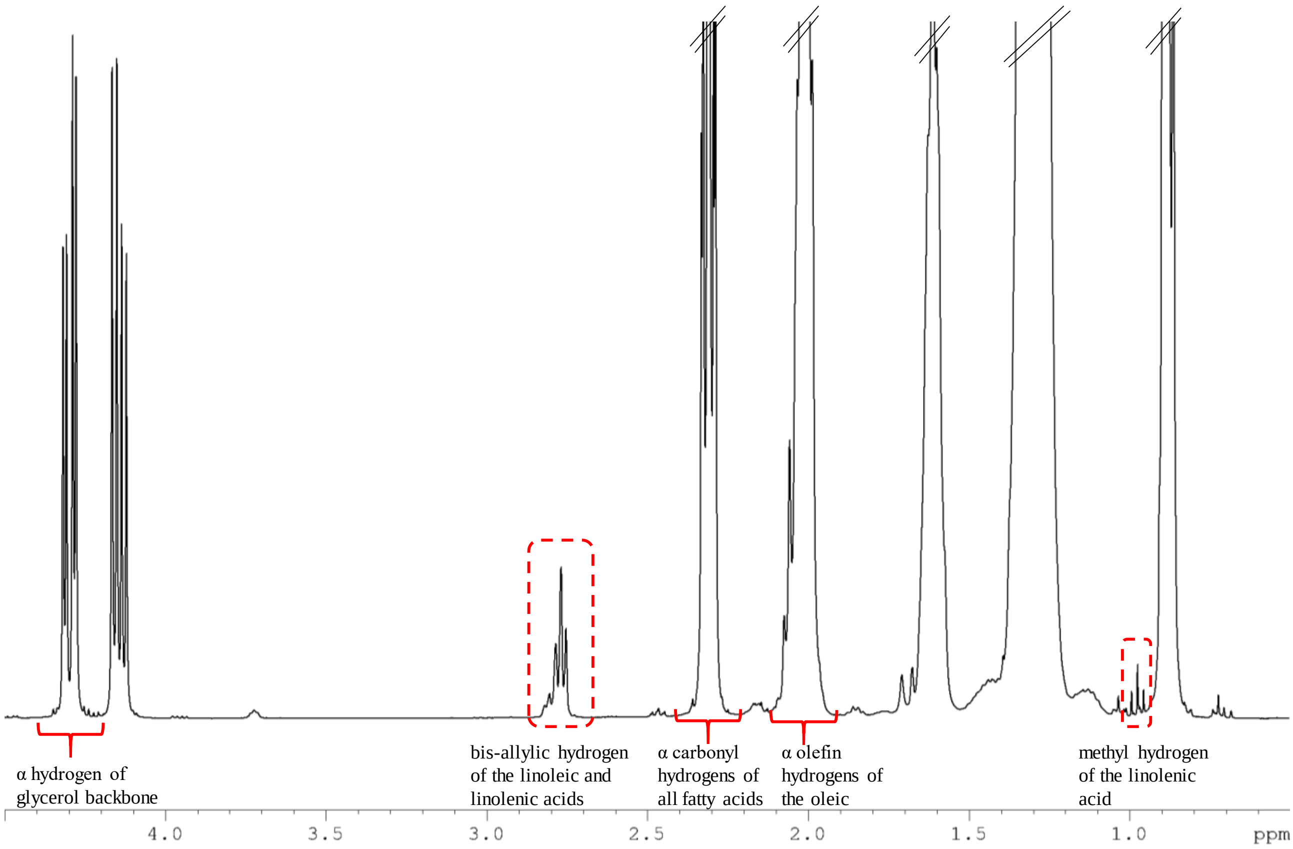

Figure S6. ^{1}H ZG NMR spectrum of olive oil with respective assignment of glycerol and fatty acids signals.

Table S1. Summary of the methodology developed to determine the fatty acid composition in edible oils by Barison et al.

Fatty acids	Integrated region (ppm)	Functional group	calibrated area value of glycerol *	Subtraction factor**
Linolenic acid (Ln)	0.999 - 0.949	C***H₃***	22.2	-
Linoleic acid (Li)	2.8353 - 2.7259	=HC-C***H₂***-CH=	33.3	– 2 x % Ln
Oleic acid (Ol)	2.1 - 1.9367	-C***H₂***-CH=CH-	16.7	– % (Ln+Li)
Saturated Fatty acid (SFA)	2.3531 - 2.25	-OCO-C ***H₂***-	33.3	– % (Ln + Li + Ol)

* This value refers to the ratio of two α glycerol protons to the number of protons that generate the fatty acid signal within a triacylglycerol

** - Linolenic acid was obtained directly measuring the area of the signal at 0.999 - 0.949 ppm after calibration of the glycerol signal (at 4.29 ppm) to 22.2 (This value refers to the ratio of two α glycerol protons to nine methyl protons of the linolenic converted into a percentage: 2/9 * 100).

- Linoleic acid was obtained after calibrating the glycerol signal to 33.3 (This value refers to the ratio of two α glycerol protons to six possible methylene protons between olefins from the linoleic acid converted into a percentage: 2/6 * 100). The signal area at 2.8353 - 2.7259 ppm provides directly the percentage of linoleic plus linolenic acid in olive oil samples. Since linoleic acid also contain methylene protons between olefins that contribute to the specific resonance, the linoleic acid percentage can be determined removing the linolenic acid contribution. Linoleic acid contains two methylene protons between olefins, whereas linolenic acid contains four; therefore, the amount of linoleic acid is calculated by subtracting twice the previously determined linolenic acid content from linoleic acid percentage
- Oleic acid was obtained after calibrating the glycerol signal to 16.7 (This value refers to the ratio of two α glycerol protons to 12 possible α olefin protons of the fatty acyl chains: 2/12 * 100) and measuring the area of signal at 2.1 - 1.9367 ppm, which refers to the methylene α olefin protons of all unsaturated fatty acids in the sample (oleic, linoleic and linolenic). Consequently, the oleic acid content is obtained by subtracting from the value obtained, the percentages of linoleic and linolenic acids previously calculated.
- Saturated fatty acid was obtained after calibrating the glycerol signal to 33.3 (This value refers to the ratio of two α glycerol protons to six α carbonyl protons of all fatty acids esterified to the glycerol moiety: 2/6 *100), integrating the signals at 2.3531 - 2.25 ppm, which refers to α carbonyl protons of all fatty acids esterified to the glycerol molecule. Consequently, when the signal at 4.29 ppm is calibrated to 33.3, the signal area at 2.3531 – 2.25 ppm is 100. For this reason, the percentage of SFA is determined by subtracting the value found, the percentages of all unsaturated fatty acids, in particular oleic, linoleic and linoleinc acid previously obtained.

a

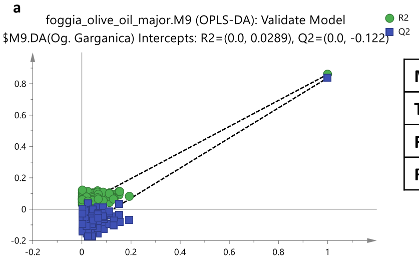

M9 (cor/og)	SS	DF	MS	F	p	SD
Total corr.	183	183	1			1
Regression	153.318	8	19.1648	112.992	0	4.37776
Residual	29.682	175	0.169611			0.411839

b

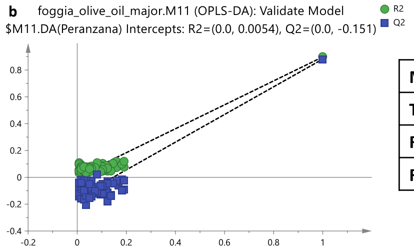

M11(cor/per)	SS	DF	MS	F	p	SD
Total corr.	182	182	1			1
Regression	160.399	8	20.0498	161.501	0	4.4777
Residual	21.6015	174	0.124146			0.352344

c

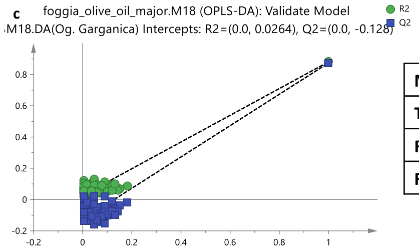

M18(og/per)	SS	DF	MS	F	p	SD
Total corr.	190	190	1			1
Regression	166.023	8	20.7529	157.529	0	4.55554
Residual	23.9767	182	0.13174			0.36296

d

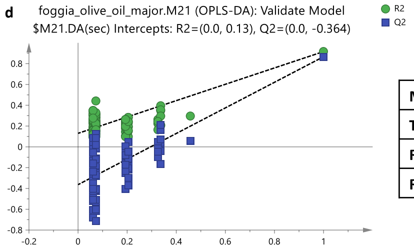

M21(Sec/young)	SS	DF	MS	F	p	SD
Total corr.	34	34	1			1
Regression	29.4668	4	7.3667	48.7516	1.04718e-012	2.71417
Residual	4.5332	30	0.151107			0.388725

Figure S7. Permutation test performed with 100 cycles of random permutation of Y variables on OPLS-DA analysis obtained for major component of olive oils (BUCKET-1). The horizontal axis shows the correlation between the original and permuted y. The vertical axis shows the values for R2 (green line) and Q2 (blue line). The intercept is a measure of the overfit. A steep slope indicates well fit. Table CV-ANOVA obtained for OPLS-DA model: **a)** Permutation test and CV-ANOVA for OPLS-DA model of olive oil samples obtained from Coratina and Ogliarola Garganica; **b)** Permutation test and CV-ANOVA for OPLS-DA model of olive oil samples obtained from Coratina and Peranzana; **c)** Permutation test and CV-ANOVA for OPLS-DA model of olive oil samples obtained from Ogliarola garganica and Peranzana; **d)** Permutation test and CV-ANOVA for OPLS-DA model of olive oil samples obtained from young or secular trees of Peranzana.

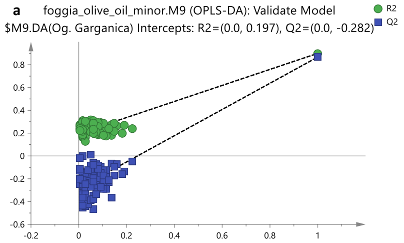

M9(cor/og)	SS	DF	MS	F	p	SD
**Total corr.**	182	182	1			1
**Regression**	157.848	8	19.731	142.147	0	4.44195
**Residual**	24.1523	174	0.138806			0.372567

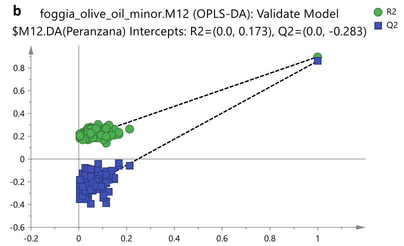

M12(cor/per)	SS	DF	MS	F	p	SD
**Total corr.**	182	182	1			1
**Regression**	156.85	8	19.6063	135.647	0	4.4279
**Residual**	25.1498	174	0.144539			0.380183

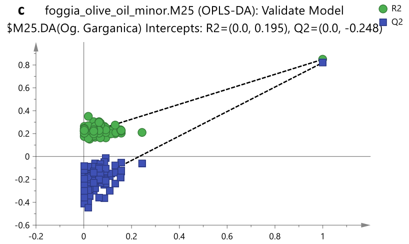

M25(og/per)	SS	DF	MS	F	p	SD
**Total corr.**	179	179	1			1
**Regression**	146.897	8	18.3621	97.8078	0	4.28511
**Residual**	32.103	171	0.187737			0.433286

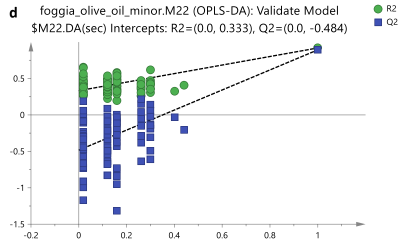

M22(Sec/young)	SS	DF	MS	F	p	SD
**Total corr.**	34	34	1			1
**Regression**	31.3803	6	5.23005	55.9003	2.68635e-014	2.28693
**Residual**	2.61969	28	0.0935603			0.305876

Figure S8. Permutation test performed with 100 cycles of random permutation of Y variables on OPLS-DA analysis obtained for minor component of olive oils (BUCKET-2). The horizontal axis shows the correlation between the original and permuted y. The vertical axis shows the values for R2 (green line) and Q2 (blue line). The intercept is a measure of the overfit. A steep slope indicates well fit. Table CV-ANOVA obtained for OPLS-DA model: **a)** Permutation test and CV-ANOVA for OPLS-DA model of olive oil samples obtained from Coratina and Ogliarola Garganica; **b)** Permutation test and CV-ANOVA for OPLS-DA model of olive oil samples obtained from Coratina and Peranzana; **c)** Permutation test and CV-ANOVA for OPLS -DA model of olive oil samples obtained from Ogliarola garganica and Peranzana; **d)** Permutation test and CV-ANOVA for OPLS-DA model of olive oil samples obtained from young or secular trees of Peranzana.

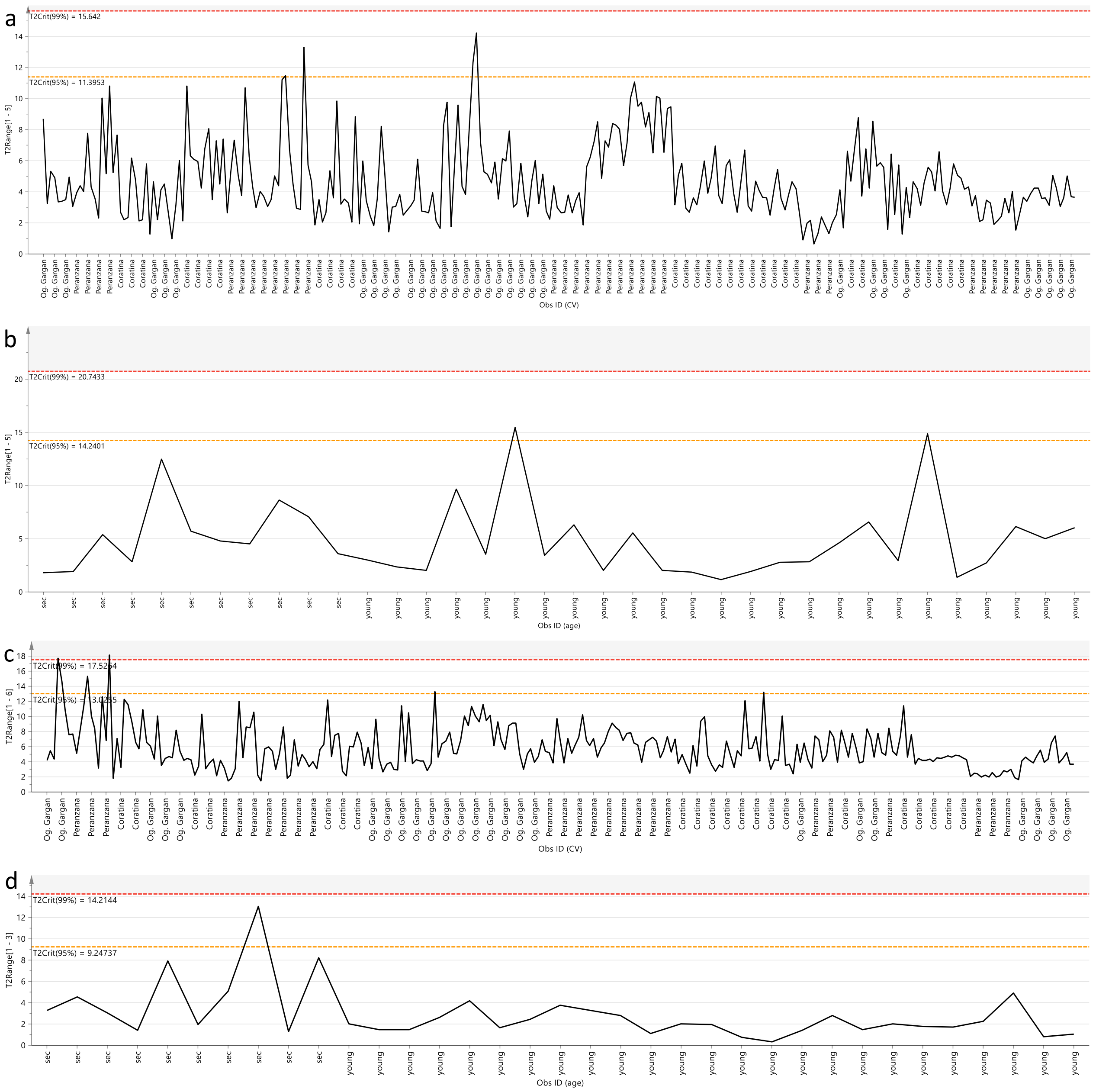

Figure S9. **a)** Hotelling's T^2 Range plot for PCA model performed on major components (BUCKET-1) of olive oil samples obtained from Coratina, Ogliarola garganica, Peranzana **b)** Hotelling's T^2 Range plot for PCA model performed on major components (BUCKET-1) of olive oil samples obtained from young and secular trees of Peranzana; **c)** Hotelling's T^2 Range plot for PCA model performed on minor components (BUCKET-2) of olive oil samples obtained from Coratina, Ogliarola garganica, Peranzana **b)** Hotelling's T^2 Range plot for PCA model performed on minor components (BUCKET-2) of olive oil samples obtained from young and secular trees of Peranzana.

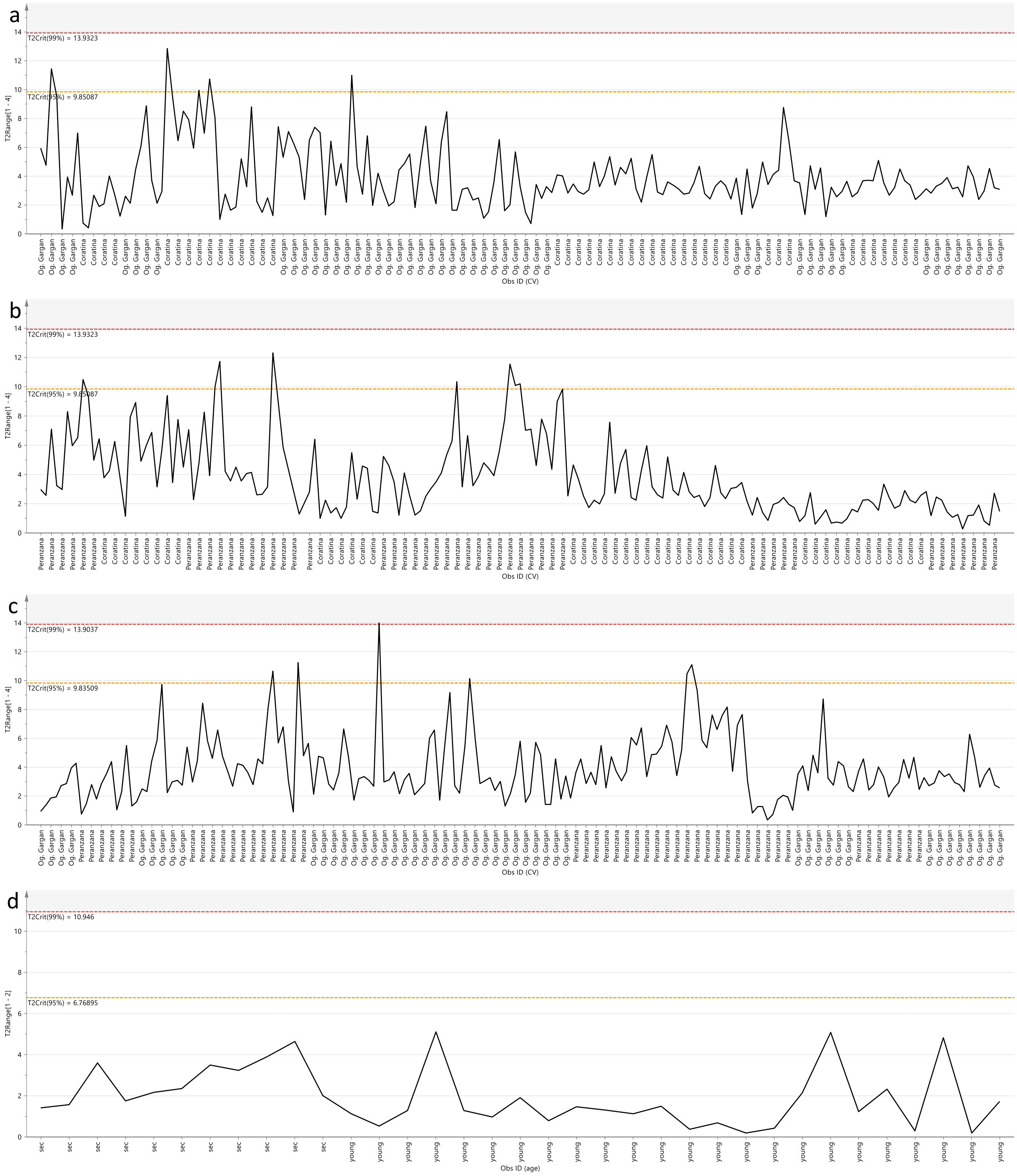

Figure S10. Hotelling's T^2 Range plot for the OPLS-DA analysis obtained for major component of olive oils (BUCKET-1) **a)** Coratina and Ogliarola garganica comparison; **b)** Coratina and Peranzana comparison; **c)** Peranzana and Ogliarola garganica comparison; **d)** young or secular trees of Peranzana.

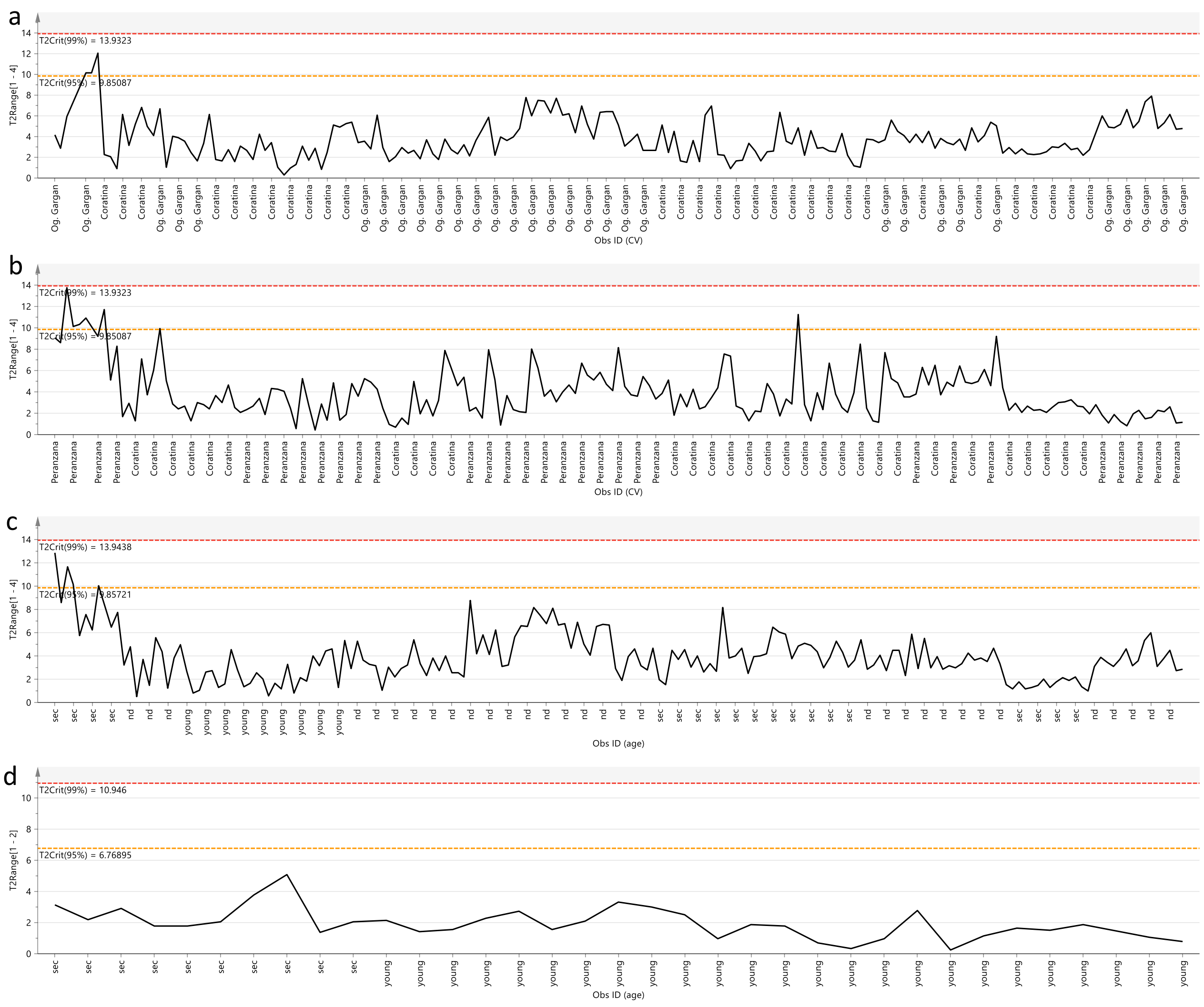

Figure S11. Hotelling's T^2 Range plot for the OPLS-DA analysis obtained for minor component of olive oils (BUCKET-2) **a)** Coratina and Ogliarola garganica comparison; **b)** Coratina and Peranzana comparison; **c)** Peranzana and Ogliarola garganica comparison; **d)** young or secular trees of Peranzana.

Table S2. Misclassification table (SIMCA-P Software Version 5) obtained for OPLS-DA model analysis performed of major component (BUCKET-1) and minor component (BUCKET-2) of olive oils obtained from young and secular trees.

	Members	Correct	sec	young	No class (YPred <= 0.65)
**Major component**					
secular	11	100%	11	0	0
young	24	100%	0	24	0
No class	0		0	0	0
Total	35	100%	11	24	0
Fisher's prob.	2.4e-009				
**Minor component**					
secular	10	100%	10	0	0
young	24	100%	0	24	0
No class	0		0	0	0
Total	34	100%	10	24	0
Fisher's prob.	7.6e-009				
